# Evaluation of Outer Membrane Vesicles Obtained from Predominant Local Isolate of *Bordetella pertussis* as a Vaccine Candidate

**DOI:** 10.52547/ibj.25.6.399

**Published:** 2021-10-31

**Authors:** Maryam Sadat Soltani, Mojtaba Noofeli, Seyed Reza Banihashemi, Fereshteh Shahcheraghi, Fereshteh Eftekhar

**Affiliations:** 1 Department of Microbiology and Microbial Biotechnology, Faculty of Life Sciences and Biotechnology, Shahid Beheshti University, Tehran, Iran;; 2 Department of Bacteriology, Pasteur Institute of Iran, Tehran, Iran;; 3 Department of Human Bacterial Vaccines Production and Research, Razi Vaccine and Serum Research Institute, Agricultural Research, Education and Extension Organization (AREEO), Karaj, Iran;; 4 Department of Immunology, Razi Vaccine and Serum Research Institute, Agricultural Research, Education and Extension Organization (AREEO), Karaj, Iran

**Keywords:** Bordetella pertussis, Vaccines, Virulence factors

## Abstract

**Background:**

Pertussis is a current contagious bacterial disease caused by Bp. Given the prevalence of pertussis, development of new vaccines is important. This study was attempted to evaluate the expression of main virulence factors (PTX, PRN, and FHA) from Bp predominant strains and also compare the expression of these factors in the OMVs obtained from predominant circulating Bp isolate.

**Methods:**

The physicochemical features of the prepared OMVs were analyzed by electron microscopy and SDS-PAGE. The presence of the mentioned virulence factors was confirmed by Western blotting. BALB/c mice (n = 21) immunized with characterized OMVs were challenged intranasally with sublethal doses of Bp, to examine their protective capacity.

**Results:**

Electron microscopic examination of the OMVs indicated vesicles within the range of 40 to 200 nm. SDS-PAGE and Western blotting demonstrated the expression of all three main protective immunogens (PTX, PRN, and FHA), prevalent in the predominant, challenge, and vaccine strains, and OMVs of the predominant IR37 strain and BP134 vaccine strain. Significant differences were observed in lung bacterial counts between the immunized mice with OMV (30 CFU/lung) compared to the negative control group ((6 10^4 ^CFU/lung; *p *< 0.001). In mice immunized with OMVs (3 µg), the number of lungs recovered colonies after five days dropped at least five orders of magnitude compared to the control group.

**Conclusion:**

OMVs obtained from circulating isolates with the predominant profile may constitute a highly promising vaccine quality. They also can be proposed as a potential basic material for the development of new pertussis vaccine candidate.

## INTRODUCTION

Pertussis or whooping cough is a current respiratory bacterial disease caused by Bp. Before the introduction of high efficient vaccines in the second half of the 20^th^ century, pertussis was recognized as a childhood disease with high mortality rates. Later, following the success of the infant immunization programs, pertussis was speculated to be under control^[^^1^^]^. Unexpectedly, during the last decade, the disease was still among the public-health problems, particularly in countries with a regular vaccination program^[^^2^^,^^3^^]^. Recently, a large number of pertussis outbreaks were observed in several countries^[^^4^^,^^5^^]^. Although it is not a life-threatening disease in adults, the affected people can act as reservoirs for the transmission of the disease among children^[^^6^^-^^8^^]^. Based on existing evidence, the resurgence of the disease is prevalent mostly in developing countries and developed world^[^^9^^,^^10^^] ^due to adaptation of pathogen populations, reduced long-term immunity of pertussis vaccine in prolonged period of time, and the use of aP instead of wP^[^^10^^,^^11^^]^.

Studies have reported genetic differences between the current circulating Bp and vaccine strains, particularly in genes encoding the vaccine component proteins, including PTX, (an AB5 toxin composed of the A [S1] subunit, and the B oligomer) and adhesions, including PRN, FHA, and fimbriae. These differences could result in inadequate immunity induction to protect against new isolates^[^^11^^,^^12^^]^. This genetic adaptation can affect bacterial protein expression, as well as vaccine-induced immunity against pertussis^[^^10^^,^^13^^,^^14^^]^.

The re-emergence of the disease has pointed out the need for the development of new vaccines that are able to overcome the disadvantages of the conventional vaccines^[^^15^^]^. To this end, the OMVs obtained from Bp are possible vaccine candidates that can successfully avoid the weaknesses of current vaccines against Bp ^[^^16^^-^^18^^]^. These vesicles naturally contain bacterial surface antigens and exhibit an acceptable level of protection. It is worth noting that the new vaccine, established based on circulating strains isolated from patients, conferred protection against circulating Bp isolates and also induced long-lasting immunity^[^^18^^-^^20^^]^. Since virulence factors have been considered as the main protective antigens to the formulation of the pertussis vaccine, studies on the expressed protein level could help to provide an insight into the pertussis resurgence problem^[^^21^^,^^22^^]^. Considering the above-mentioned data, the current study aimed to evaluate the expression of main virulence factors, including PTX, PRN, and FHA from Bp predominant strains and compare to the expression of these factors in the OMVs obtained from predominant circulating Bp isolate. In addition, the physicochemical properties of OMVs, as a probable vaccine candidate, were assessed.

## MATERIALS AND METHODS

**Bacteria and culture medium**

Bacterial strains used in this study consisted of four predominant isolates (IR37, IR77, IR124, and IR148). The challenge strain (18283) and the vaccine strain (BP134) were obtained from the Pertussis Reference Laboratory at Pasteur Institute of Iran (Tehran) and the RVSRI, respectively. The bacteria were cultured on BGA supplemented with 10% defibrinated sheep blood^[^^23^^,^^24^^]^.

**Isolation of OMVs and preparation of growth curve**

OMWs were obtained from bacterial cells. Briefly, bacteria were sub-cultured into MSS liquid medium containing methyl-β-cyclodextrin at 36 °C with aeration at 150 rpm (HYSC, Korea)^[^^24^^]^. Since the time of OMV harvesting is very crucial for the product yield, it is important to determine the bacterial growth curve before OMV extraction. For this purpose, after 21-h incubation at 3 ×g and 36 ºC, the culture was inoculated into a fresh MSS medium at OD_600_ = 0.05, 3×g and 36 ºC. Thereafter, the samples were collected continuously at fixed intervals for 70 h to plot the growth curve. At the end of the logarithmic phase, (OD_600_ = 0.75-1), 10 ml was inoculated into a 500-ml fresh MSS medium with initial turbidity at OD_600_ = 0.05 and then incubated at 3 ×g at 36ºC for 34 h^[^^25^^]^. OMVs were prepared as previously described with modifications to avoid the need for ultracentrifuge with high speed^[^^24^^-^^26^^]^. Briefly, the bacterial strains were pelleted from MSS (at 8000 ×g at 4 °C for 30 min). The pellets were washed with TE and centrifuged, and then the supernatants were pelleted at 60,000 ×g at 4 °C for 2 h. Subsequently, these resulting pellets were resuspended in a TE buffer containing deoxycholate (5 g/L) and mixed again several times by pipetting to make a homogenized suspension. The mixture was incubated for 10 min and then centrifuged at 60,000 g at 4 °C for 2 h. Afterward, the supernatant was separated carefully in a new tube and treated with TE buffer and centrifuged again at 60,000 ×g at 4 °C for 1 h. The pellets were suspended in 6 ml of 3% sucrose and filtered with 0.22-µm pore size filters (polyvinylidene difluoride, syringe filters, Sigma-Aldrich, Germany)^[^^24^^,^^27^^]^. The OMV sample was inactivated in a bain-marie (Memmert, Germany) at 56 °C for 30 min. The sterility and viability of the samples were controlled by incubating the suspension on BGA and blood agar plates. The OMVs were examined by TEM^[^^24^^-^^26^^]^.

**Gel electrophoresis and Western blot analysis**

The four predominant strains, challenge strain, vaccine strain were grown on BGA. A loop full of Bp bacteria from a 48h-old culture on BGA was taken and resuspended in 1 ml of PBS and incubated at 56 °C for 30 min. The suspensions were then diluted in a sample buffer and boiled for 15 min before being subjected to electrophoresis^[^^26^^]^, In addition, OMVs (5 µg) obtained from predominant and vaccine strains were resuspended in a sample buffer, boiled by heating at 100 °C for 10 min and run on 10% SDS gels^[^^24^^]^. Electrophoresis was performed at RT with constant voltage of 80 V. Polypeptides were stained with a solution of Coomassie Blue R250 (DNA biotech, Iran) with gentle shaking for 1 h. The gels were then washed three times in methanol 40% (v/v) and acetic acid 10% (v/v) in double distilled water for 20 min^[^^28^^]^. For Western blot analysis, the separated protein bands were transferred from polyacrylamide SDS gels to a polyvinylidene difluoride membrane. The membranes were then blocked with 5% skim milk in PBS overnight, washed three times with PBS in 0.05% Tween 20 and incubated with PRN, FHA, and PTX monoclonal antibodies (NIBSC No. 97/558, 97/564, and 97/572, respectively) at RT for 1 h. The washing steps were repeated thrice, followed by incubation with horseradish peroxidase-conjugated rabbit anti-sheep antibody (PADZA Company, Iran) at a 1:1000 dilution, at RT for 1 h. After additional three times washing with PBST, the membranes were developed with Metal Enhanced DAB Substrate kit (Sigma-Aldrich)^[ ^^28^^]^.

**LAL test**

The LPS toxicity content in OMVs was determined by the LAL test. The chromogenic LAL assay (Thermo Fisher Scientific, USA) was performed to determine the presence of LPS and measure the endotoxins in the samples^[^^29^^,^^30^^]^.

**Animal model**

Female BALB/c mice at the age of 6-8 weeks old (weighing 15-20 g) were provided by RVSRI. In order to be adapted to the environment, all the animals were kept under standard conditions one week before the experiments.

**MWG test**

The safety of OMVs obtained from predominant strain IR37 was determined by the MWG test, according to the WHO guidelines^[^^31^^]^. A group of eight BALB/c mice were injected intraperitoneally with detoxified OMVs acquired from circulating strain with predominant profile (IR37). OMVs were detoxified with 0.4% formalin. Two different concentrations of OMVs (3 µg and 20 µg in 40 µl PBS) were used in this experiment, along with PBS as negative control. The weight of mice was measured at specific intervals (16 h, three and seven days). The non-toxicity of extracted OMVs were confirmed by passing the WHO requirement as follows: (1) the total weight of the mice from the vaccine group three days after injection was higher than or same as the initial weight; (2) the weight gain average was compared with the control group at the end of the day seven, which should not be less than 60% of the control group, based on the WHO guidelines; (3) more than 95% of the animals survived during the test. The side effects of the injection site and the rate of mortality were examined, as well. ANOVA test was used to compare the data obtained^[^^28^^,^^31^^,^^32^^]^.

**Immunization regimen**

To evaluate the immunological responses to OMVs obtained from the predominant strain IR37 of Bp*, *vaccination was carried out in three groups (seven mice per group). Each immunization dose for injection comprised 3 µg of protein in 40 µl of PBS, according to the result of the MWG test^[^^32^^]^. The mice were injected with OMVs, PBS as negative control, and commercial Tdap vaccine (ADACEL, Sanofi Pasteur, France; 1:10 of human equivalent dose) as the positive control. All the mice were immunized intraperitoneally on days 0, 14, and 28^[^^27^^,^^32^^]^.

***B. pertussis*** **challenge**

To evaluate the immunization efficacy, the mice were challenged with the intranasal injection of sublethal dose challenge strain 18323) 1.4 × 10^7 ^Bp*(* two weeks after the last immunization. For bacterial count, the lungs of the injected mice were collected after seven days. The lungs were homogenized in the sterile PBS and were diluted, and then cultured on BGA containing 10% defibrinated sheep blood to determine bacterial recoveries. The experiments were performed three times^[^^27^^,^^31^^,^^32^^]^.

**Statistical analysis**

Standard deviations and means were calculated from Log10-transformed CFU numbers. Differences between means were analyzed by one-way ANOVA with statistical significance accepted at the *p*< 0.05 level^[^^28^^]^. 

**Ethical statement**

All animal experiments, including toxicity MWG test, immunization, and challenge test were approved by the Ethical Committee of Animal Care of RVSRI and Shahid Beheshti University (Ethical code: IR.SBU.REC.1398.014).

**Fig. 1:**
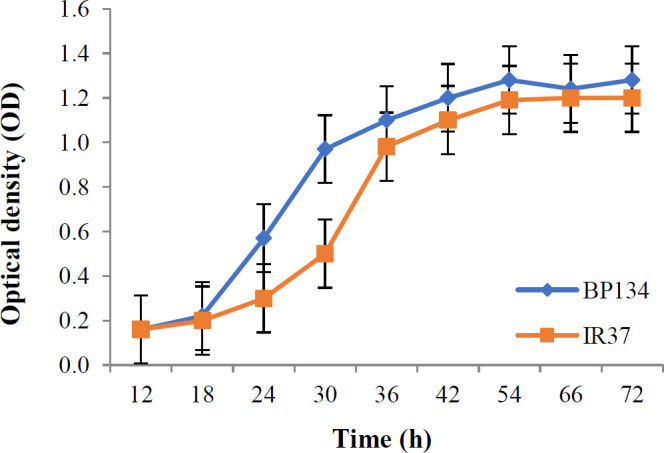
The growth curve of the circulating strain BP IR37 and vaccine strain BP134. Inoculations were carried out in triplicate, and the OD was recorded at 600 nm

## RESULTS

**Fig. 2:**
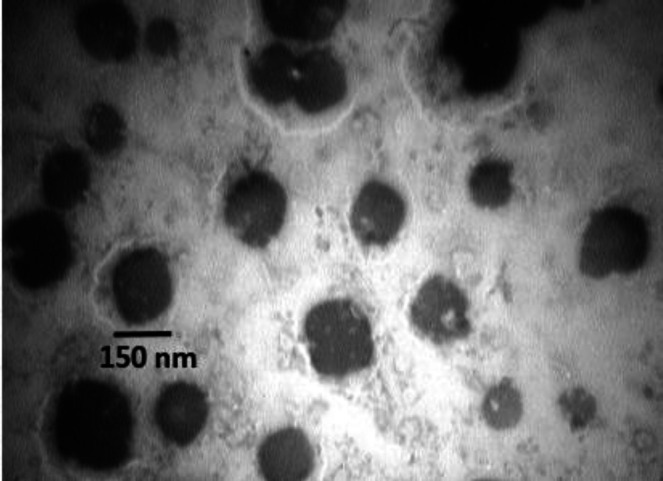
Negatively stained *Bordetella pertussis *OMVs examined with an electron microscope

In liquid cultures, various parameters including medium composition, stress conditions, and cell harvest time, affect the amount of bacterial OMVs vesiculation. Considering the importance of cell harvest time in the process of OMV preparation, our result showed that the optimum time for collecting OMVs would be the end of the logarithmic phase, as in this time point the bacterial growth will be stopped and t the OMV extraction process started. The turbidity of liquid culture was approximately 0.7 to 1 at 600 nm wavelengths, which was about 30 h after the initial culture for vaccine strain and about 36 h for predominant strain IR37 (Fig. 1)^[^^25^^]^. The OMVs were obtained from predominant strain IR37 and vaccine strain BP134 as previously described. The electron microscopic examinations indicated that the mean size of extracted OMVs were 70 nm and ranged from 40 to 200 nm (Fig. 2). The OMV extraction procedure repeated five times, and in all cases the results were similar. 

**Fig. 3:**
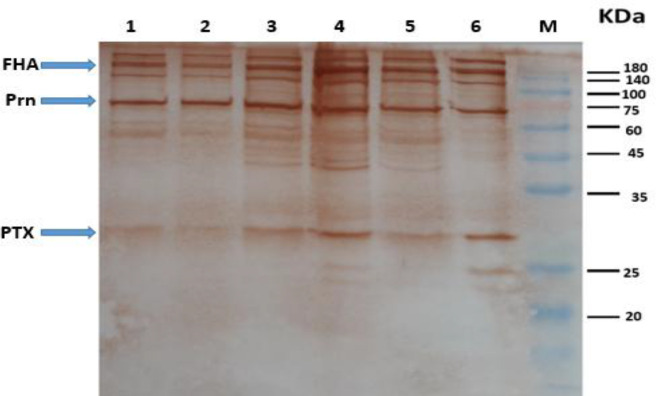
Western blot of samples. Lane 1-4, predominant strains (IR148, IR77, IR124, and IR37); lane 5, challenge strain; lane 6, vaccine strain; M, molecular marker

**Fig. 4:**
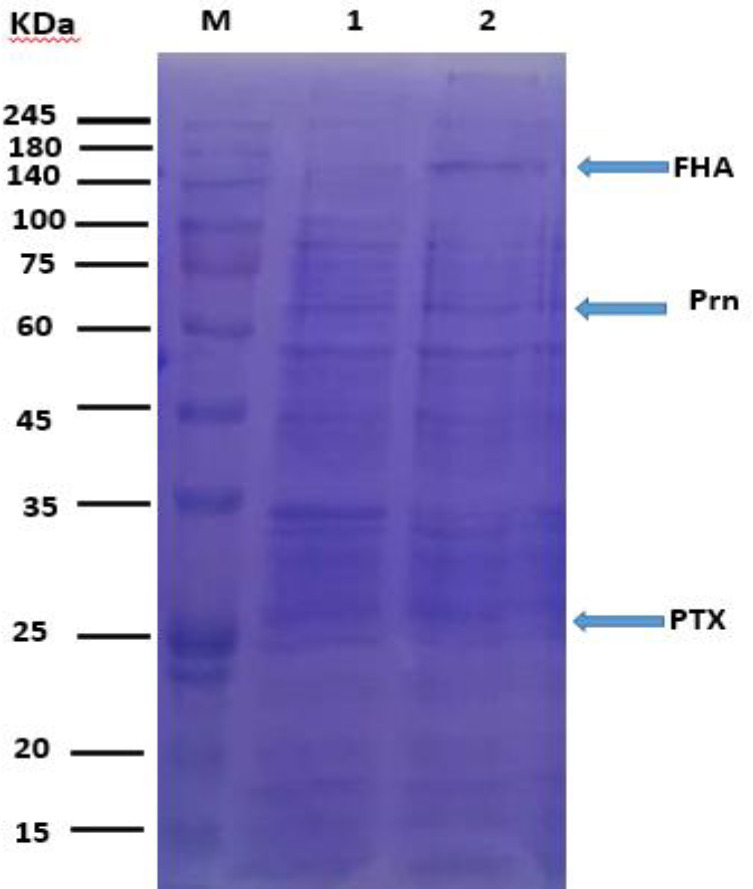
The SDS-PAGE pattern of OMVs containing FHA, PRN, and PTX. Lane 1, the OMV obtained from vaccine strain; lane 2, the OMV obtained from predominant strain IR37; M, molecular marker

**Fig. 5:**
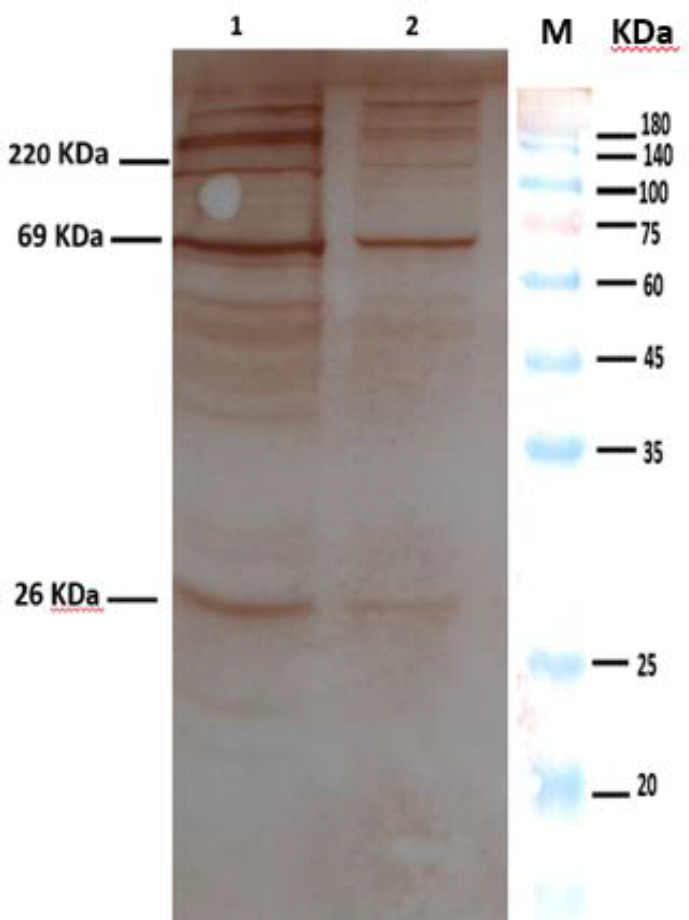
Western blot of OMVs using anti-FHA, anti-PRN, and anti-PTX. Lane 1, the OMV obtained from vaccine strain; lane 2, the OMV obtained from predominant strain IR37; M, molecular marker

Samples of bacteria, including the four predominant strains, challenge strain, vaccine strain and OMV, were obtained from predominant strain. Vaccine strains were screened by SDS-PAGE and Western immunoblot for the expression of PTX, PRN, and FHA. Western immunoblot results showed the presence of all three main protective immunogens in all predominant strains, challenge strain, vaccine strain, and also in the OMVs of predominant strain IR37 and OMVs of vaccine strain BP134 (Figs. 3-5).

The LPS toxicity of the OMV final lot samples was tested in a fivefold dilution and contained 15,000 EU/ml. The endotoxin activity was within the range of DPT/polio vaccines and is therefore regarded as safe^[^^31^^]^. The MWG test is used to evaluate the toxicity of the injected OMVs. According to Table 1, the extracted OMVs were injected with a lower dose of 3 μg, which showed no AT, no significant weight loss of the mice, and no abnormal local tolerance at the injection site, and all mice survived during the experiment period. Therefore, it means the amounts of OMVs applied were non-toxic based on the WHO criteria for the MWG test^[^^31^^]^. However, the results of higher dose (20 µg) showed weight loss three days post vaccination. According to these observations, the safety dose of OMVs was about 3 µg. This amount of OMVs considered for further examination.

**Murine challenge**

**Fig. 6:**
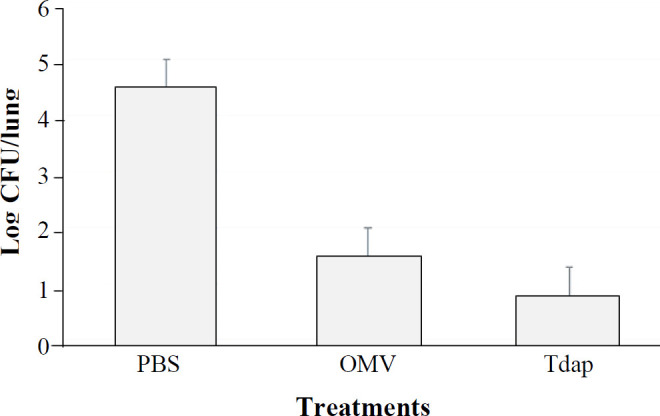
Comparison of the lung clearance efficiency of OMVs with Tdap and PBS

Based on the immunization program, seven BALB/c mice per group were immunized either by OMVs extracted from predominant strain or positive control, as well as negative control., The immunized mice were then challenged with IR37 strain intranasally at a concentration of 1.4 × 10^7^, two weeks after immunization. The survival time was also monitored for the next 10 days (Fig. 6). As expected, significant differences in lung bacterial counts between the control group and immunized mice were observed (*p *< 0.001). In mice injected with PBS, the number of recovered colonies was 6 10^4^ CFU/lung, while in mice immunized with OMVs (3 µg), the number of recovered colonies (30 CFU/lung) after five days dropped at least 3 orders of magnitude in comparison to the negative control group.

## DISCUSSION

Currently, there are two different kinds of pertussis vaccines. The wP that is the first generation vaccine developed in the 1940s and comprises of killed bacteria. Despite the reactogenicity and side effects, this type of vaccine has resulted in a significant decrease in pertussis incidence. However, the above mentioned drawbacks encouraged the researchers to introduce the second generation pertussis vaccine, namely aP, which was developed in the early 1990s. The aP vaccines include three to five antigens purified from Bp and are broadly used around the world; however, these vaccines have some disadvantages such as low efficacy and short-term immunity^[^^33^^,^^34^^]^.

**Table 1:** Toxicity test (mouse weight gain test)

**Dose of infection**	**Average weight gain per mouse (g)**		
	**Day 3**	**Day 7**	**Weight gain compared to the control (%)**
3	0.8	1	74
20	-1.4	-0.5	-32
PBS	0.6	1.4	----

The average weight gain per mouse was determined after three and seven days after intraperitoneal injection.

Despite vaccination against pertussis with a high rate of coverage, there are concerns regarding the recurrence of the disease^[^^2^^,^^21^^]^. Among the possible explanations for the re-emergence of the disease, pathogen adaptation is one of the main issues. Investigations focusing on pathogen adaptation have reported the antigenic divergence between the isolates used for vaccine development and the currently circulating isolates in different regions, affecting the expression of virulence factors^[^^3^^,^^35^^]^. This antigenic divergence commonly occurs during the bacterial genome evolution to escape from the immune system, which increases the adaptation of the bacterial population. It leads to waning the efficacy of the vaccine-induced immunity against the new isolates over the time and decreases the period in which pertussis vaccine is effective^[^^36^^,^^37^^]^. 

In a study performed by Fry *et al.*^[^^38^^]^on the allelic shift of the PTX gene, *ptxA1* had the most allelic diversity. Similar allelic diversity in different countries has been reported, as well. Herein, the *ptxA1*, which was different from the vaccine strain alleles, was the predominant circulating allele in different countries. Mooi *et al.*^[^^35^^,^^39^^,^^40^^] ^demonstrated polymorphisms in vaccine antigen genes such as *ptxP*,* ptxA*,* prn*, and *fim*. 

In Iran, the disease incidence has been increasing despite the high rate (more than 96%) of wP vaccination using the same strains in vaccines without any changes during the last 60 years^[^^41^^]^. According to previous studies conducted in Iran, the allelic profiles of predominant circulating isolates were* ptx*A1, *ptx*P3, *prn*2, *fim*2-1, and *fim*3-2. It is important to note that this pattern is not completely equivalent to the vaccine strain BP134 profiles *ptx*P1, *ptx*A2, *prn*1, and *fim*3-2^[^^41^^-^^43^^]^. Moreover, The presence of *ptxP3*, *ptxA1*, and *prn2 *alleles results in enhancing the capacity of the strain to spread among the immunized populations^[^^12^^,^^44^^]^. Another genomic change in Bp isolates has been the deletion of the PRN gene. PRN is the main antigen component of the pertussis vaccines, and those Bp strains that lack the expression of PRN protein are called PRN negative^[^^45^^,^^46^^]^. Therefore, it was important to evaluate the presence of PRN and other virulence factors in the native predominant strains.

In this research, some circulating isolates with predominant profiles were selected from the Biobank of the Pasteur Institute of Iran, which based on Western blot results, all expressed PTX, PRN, FHA similar to vaccine strains and challenge strain (Fig. 3). Despite the presence of main protective antigens to overcome the drawbacks related to the conventional vaccines, using predominant circulating strains in vaccine might be effective to decrease the incidence of pertussis and potentially eradicate Bp worldwide^[^^47^^,^^48^^]^. In this regard, the use of predominant strain in vaccine development will be a first step forward to control pertussis worldwide.

One of the drawbacks of aP pertussis vaccines is decreased vaccine efficacy, due to the waning vaccine immunity by the rapid reduction of antibodies following vaccination and long-term immunity^[^^11^^]^. Although aP vaccines provide immunity against specific antigens used in the vaccine formulation, wP vaccines provide immunity against the most common antigens of bacterial whole cells^[^^11^^,^^49^^]^. aP vaccines also elicit humoral immune response Th2/Th17. Because Bp is an intracellular bacterium, the cellular immune response Th1/Th17 plays a main role in the clearance of the bacteria from the respiratory tract. Moreover, the humoral response is an insufficient immune response needed for complete protective immunity against pertussis^[^^49^^,^^50^^]^. Thus, studies have attempted to develop the third generation of new protein-based vaccines, such as OMVs vaccines, which besides having several immunogenic proteins, they can induce Th2 , Th1, and Th17 responses^[^^50^^,^^51^^]^. A significant alternative protective subunit is the OMV produced by the Gram-negative bacteria as a natural budding element, which can be obtained by detergent extraction from Bp^[^^52^^]^. The component of the OMVs is complex, which the main constituents include outer membrane proteins, periplasmic proteins, phospholipids, lipopolysaccharides, and nucleic acids. The interaction of the above components with each other determines the physicochemical properties of OMV and its stability^[^^17^^]^.

The size range of OMVs also indicated that OMVs were nanoparticles that can be taken up by the antigen-presenting cells^[^^53^^]^. OMVs act as pathogen-associated molecular patterns, which are detected by the immune system and elicit the innate immune response and have adjuvant activity, as well^[^^54^^]^. The OMV vaccines can be considered effective and also safe vaccines^[^^17^^,^^55^^]^. The first attempts to apply OMVs in the field of vaccines were in response to the serogroup B meningococcal disease by the Finlay Institute in Cuba^[^^56^^]^.

In recent years, numerous studies around the world have evaluated the potential use of OMVs, as vaccine candidate for pertussis^[^^16^^,^^17^^,^^19^^,^^27^^,^^54^^]^. Selective pressure on circulating strains cannot affect OMV contents, therefore making the emergence of escape isolates more difficult, as was observed for pertussis aP vaccines made with purified protein antigen^[^^17^^,^^51^^]^.

In this study, OMVs were obtained from Bp strain IR37 with predominant genomic profile and also vaccine strain BP134. OMVs were extracted by the modified method to avoid the need for ultracentrifuge with high speed, which is expensive and is not generally available in laboratories and many research centers^[^^26^^]^. The TEM result indicated that the extracted OMVs were stable in conformation, the same as previous studies^[^^19^^,^^27^^]^. 

Since OMVs have the ability to induce immune response as an immunogen, due to carrying several important antigens, the presence of the main virulence factors such as PTX, PRN, and FHA in the OMVs is very important^[^^16^^,^^27^^]^. In this context, we investigated the expression of the PTX, PRN, and FHA in OMVs obtained from predominant Bp and vaccine strains. Our results confirmed the existence of these antigens in investigated OMVs. The presence of surface immunogens such as PTX, PRN, and FHA allowed us to verify that the OMVs obtained from the outer membrane, as described before^[^^16^^]^. The OMV extraction procedure repeated five times, and in all cases, the same results were observed, which was similar to the previous studies^[^^18^^,^^27^^]^. Furthermore, the presence of main virulence factors was detected in all samples. The safety of using these extracted OMVs was evaluated by the abnormal toxicity test. The LAL test results showed that the extracted OMVs were safe^[^^30^^]^. The MWG test, which is often used to determine the toxicity of the injected OMVs, using low dose (3 μg), showed no difference in the weight gain curve, meaning that this amount of OMVs passed toxicity criteria successfully and considered as non-toxic. This finding is in agreement with former studies^[^^19^^,^^32^^]^. The results indicated that the samples were not contaminated with impurities during the OMV preparation process.

In order to determine the protection capacity induced by extracted OMVs, animal studies using intranasal Bp challenge were conducted. Adequate clearance rates (*p*< 0.005) were detected in mice immunized with extracted OMVs and commercial Tdap as a positive control. However, in mice injected with PBS (as negative control), the number of recovered colonies was high ((6 10^4 ^per lung).). Our results showed statistically significant differences between immunized animals with OMVs and the control group (*p *< 0.001), which is comparable to other studies^[^^18^^,^^27^^,^^32^^]^.

In conclusion, according to OMVs features and due to the data presented herein and previous studies, it can be suggested that OMV-based vaccines derived from circulating isolates with the predominant profile may constitute a very promising vaccine quality. Moreover, it could be proposed as a potential basic material for developing the new generation of pertussis vaccine candidate and could be successful in the disease control. 

## CONFLICT OF INTEREST.

None declared.
